# Pharmacodynamic effects of molidustat on erythropoiesis in healthy cats

**DOI:** 10.1111/jvim.16827

**Published:** 2023-11-23

**Authors:** Annette Boegel, Ingo Flamme, Ralph Krebber, Terry Settje, Franziska Schmidt, Eva Kruedewagen, Sandra Mangold‐Gehring, Chantal Lainesse, Andreas Moritz, Gerald Beddies

**Affiliations:** ^1^ Elanco Animal Health Leverkusen Germany; ^2^ Bayer AG Leverkusen Germany; ^3^ Olathe Kansas USA; ^4^ Integral Consulting Strategies, Inc. Saskatoon Saskatchewan Canada; ^5^ Justus‐Liebig University Giessen Germany

**Keywords:** anemia, erythropoietin, hematocrit, hypoxia‐inducible factor, molidustat

## Abstract

**Background:**

Inhibition of hypoxia‐inducible factor prolyl hydroxylase (HIF‐PH) stimulates erythropoiesis in rats, dogs, monkeys, and humans.

**Hypothesis/Objective:**

Determine if molidustat, a novel HIF‐PH inhibitor, stimulates erythropoiesis in healthy cats.

**Animals:**

Seventeen healthy adult laboratory cats.

**Methods:**

Randomized, placebo‐controlled study. Cats were treated PO once daily with suspensions of 0 (Group 1; n = 6), 5 (Group 2; n = 6), or 10 (Group 3; n = 5) mg/kg of molidustat. Effects on red blood cell parameters, reticulocyte indices and plasma erythropoietin (EPO) concentrations were evaluated. Molidustat treatment was stopped when hematocrit (HCT) exceeded 60%.

**Results:**

Compared to placebo, a significant increase in mean HCT was evident starting on Day 14 (Group 2:54.4% vs 40.3%, *P* < .001, 95% confidence interval [CI] for the difference [8.95‐19.28]; Group 3:61.2% vs 40.3%, *P* < .001, 95% CI [15.48‐26.43]) and remained significantly higher for the entire treatment period. In molidustat‐treated groups, HCT exceeded 60% on Day 21 (Group 2) and Day 14 (Group 3). Mean HCT in molidustat‐treated cats returned to within the reference range (29%‐45%) after Day 56 and was numerically comparable to placebo from Day 70 onwards. Red blood cell count and hemoglobin concentrations followed a similar pattern as HCT. Mean EPO concentrations significantly increased after molidustat administration on all assessment days. Molidustat treatments were well tolerated.

**Conclusions and Clinical Importance:**

Marked erythropoietic effects were identified after daily administration of molidustat to healthy cats and additional studies are warranted to evaluate the effects in anemic cats.

**Abbreviations**
bw: body weight
CHr: reticulocyte hemoglobin concentration mean
CI: confidence interval
CKD: chronic renal disease
ELISA: enzyme‐linked immunosorbent assay
EPO: erythropoietin
ESA: erythropoietin stimulating agent
Hb: hemoglobin
HCT: hematocrit
HIF: hypoxia‐inducible factor
HIF‐PH: hypoxia‐inducible factor prolyl hydroxylase
HPLC/MS‐MS: high‐performance liquid chromatography with tandem mass spectrometric detection
PK: pharmacokinetic
RBC: red blood cell
REP: renal erythropoietin‐producing

## 1 INTRODUCTION

Erythropoiesis is an intricately coordinated homeostatic process regulated by the glycoprotein erythropoietin (EPO) predominantly secreted by specialized renal interstitial fibroblasts, called renal EPO‐producing cells that promote proliferation and differentiation of erythroid progenitor cells into red blood cells (RBCs) in the bone marrow.^1^, ^2^, ^3^, ^4^ Production of EPO is controlled by hypoxia inducible factor (HIF) degraded under normal physiological oxygen concentrations by HIF prolyl hydroxylase (HIF‐PH) oxygen‐sensing enzymes, giving mammals the ability to adapt to environmental changes in oxygen concentrations.^1^, ^2^, ^3^, ^4^ Hypoxia inducible factor is a heterodimeric transcription factor consisting of α‐ and β‐subunits binding to hypoxia inducible genes.^1^, ^2^, ^3^ Under hypoxic conditions, EPO production is increased by inhibition of HIF‐PH enzymes.^1^, ^3^ Stabilization of HIF‐α by administration of a HIF‐PH inhibitor in rats and monkeys had a dose‐dependent effect on production of EPO and erythropoiesis.^1^ Several clinical trials in humans confirmed the erythropoietic effect of HIF‐PH inhibitors in anemic patients with chronic kidney disease (CKD) by observation of increasing hemoglobin (Hb) concentrations comparable to those observed with administration of recombinant EPO.^1^, ^3^, ^4^, ^5^ It was therefore hypothesized that a HIF‐PH inhibitor also could increase EPO production and relevant hematologic parameters in healthy cats under normoxic conditions.

Our aim was to investigate the effects of repeated daily PO administration of molidustat sodium, a HIF‐PH inhibitor, on hematologic parameters and EPO concentrations in healthy adult cats.

## 2 MATERIALS AND METHODS

### 2.1 Study design

Ours was a prospective, randomized, placebo‐controlled, open‐labeled laboratory study. The study design and experimental procedures were approved by the responsible authorities and executed according to local animal welfare laws and company procedures. It was designed to investigate the pharmacodynamic effects of molidustat on erythropoiesis when administered PO once a day in a suspension at 2 different dosages in healthy adult cats in a controlled laboratory setting. In addition, plasma concentrations of molidustat were measured over time.

### 2.2 Study inclusion

Seventeen adult healthy laboratory cats (1‐2 years old; 3.0‐5.5 kg; both sexes; neutered or spayed) from the test facility's cat colony were enrolled in the study. Cats with no abnormal findings on routine serum biochemical profiles on Day −14 and hematologic analysis on Days −14 and − 7 were included.

Cats were acclimated to conditions of the study facility for at least 14 days before the study start and group‐housed in consideration of animal welfare. All cats were returned to the facility colony after study completion.

Considering a physiological hematocrit (HCT) range of 29% to 45% in cats,^6^, ^7^ treatment was intended for up to Day 56 but was ceased if HCT reached or exceeded 60% because of animal welfare concerns to avoid micro‐thromboembolic events as previously reported in dogs.^8^

Selected cats were blocked by sex and stratified by HCT on Day −7 for randomized allocation to 1 of the 3 study groups ensuring equal sex assignment.

The study was performed as an open study because of its exploratory nature and the use of quantitative assessment of pharmacodynamic effects of molidustat.

### 2.3 Treatment phase and study procedures

Cats were treated PO under fasting conditions (at least 16 hours) once daily with suspensions of molidustat sodium at 0 (Group 1; n = 6), 5 (Group 2; n = 6) or 10 (Group 3; n = 5) mg/kg body weight (BW) via single‐use syringes. Cats were fed a commercial feline dry diet (Josera Optiness, Josera GmbH, Kleinheubach, Germany) approximately 4 hours after treatment each day. Three different PO suspensions were used containing 0 (vehicle), 5% (54.6 mg/mL) and 10% (104.7 mg/mL) molidustat sodium to accommodate 3 different dosages and to standardize the administered volume to 0.1 mL/kg BW.

Physical examinations were performed bi‐weekly. Clinical observations, food consumption and fecal consistency were recorded once daily from Days 0 to 104. All cats were weighed weekly throughout the study. Blood for serum biochemistry was planned to be collected on Day −14, at the end of the treatment phase (Day 56) and again on Day 97 for all cats. Safety and tolerance of the 3 different treatments were assessed by clinically relevant findings and occurrence of adverse events. Any events of vomiting that occurred within 4 hours of treatment were considered treatment related.

### 2.4 Hematologic parameters

For evaluation of hematologic parameters, blood samples were collected at regular intervals before, during and after the treatment phase once a week up until Day 98 and analyzed using an automated hematology system (Advia 120 Hematology System, Siemens Healthcare Diagnostics, Tarrytown, USA), with a focus on RBC parameters such as HCT, RBC count, hemoglobin concentration (Hb), reticulocyte Hb concentration mean (CHr) as well as total reticulocyte count.

### 2.5 Molidustat and EPO concentrations

Blood samples were collected by venipuncture on Days 0, 7 and 23, and analyzed for plasma concentrations of molidustat (before, 2 and 24 hours after administration) and EPO (before, 6 and 24 hours after administration). Blood was collected into lithium‐heparin Monovetten sampling tubes (Sarstedt AG & Co., Nümbrecht, Germany). For plasma separation, blood samples were centrifuged at 4 to 8°C and 4000 rpm (3220×*g*) for 10 minutes and plasma was transferred to micro test tubes and stored frozen at −18°C for up to 4 weeks until transferred to the analyzing laboratory. Analysis of EPO concentrations (in milliunits per mL [mU/mL]) was performed using a combination of 2 different monoclonal antibodies provided by EPO enzyme‐linked immunosorbent assays (ELISAs) used in humans from R&D Systems (Minneapolis, USA) as capturing antibody and Roche Molecular Biochemicals (Mannheim, Germany) as detection antibody, respectively, and mouse EPO as a standard, as previously described.^9^ Bioanalysis of plasma molidustat concentrations (μg/mL) was performed at Bayer CropScience AG using an extraction followed by high‐performance liquid chromatography with tandem mass spectrometric detection (HPLC‐MS/MS) using a Zorbax Eclipse Plus C18 HD column (Agilent Technologies, Böblingen, Germany) and Sciex API 4000 mass spectrometer (Sciex, Darmstadt, Germany). A liquid extraction was performed, and the mobile phase consisted of a solution of acetonitrile/water, ammonium formate and formic acid. The lower limit of quantitation was 5 μg/L.

### 2.6 Statistical analysis

A repeated measures analysis of covariance was used to analyze RBC counts, Hb and HCT results for the treatment phase. Measurements taken before treatment were used as covariates. The best‐fitted covariate structure was used. A significant treatment by day interaction was observed in all analyses and thus pair‐wise comparisons between the treated groups vs the control group were performed for each time point. Statistical analysis of plasma EPO concentrations was performed in the same manner. Pairwise comparisons of significant treatment by day effects were performed. P‐values < .05 were considered significantly different.

Mean plasma concentrations (±SD) for molidustat and EPO were calculated from individual results for each study group.

Pre‐ and post‐treatment physical examinations and daily clinical observations were evaluated but not statistically analyzed. Additionally, BW underwent descriptive statistical evaluation.

## 3 RESULTS

On Day 14, HCT fulfilled the pre‐determined criteria for termination of treatment (≥60%) for 2 Group 3 cats and on Day 21 for 3 Group 2 cats. Molidustat treatment therefore was stopped on Day 15 for the entire 10 mg/kg group and on Day 23 for the entire 5 mg/kg group.

**FIGURE 1:**
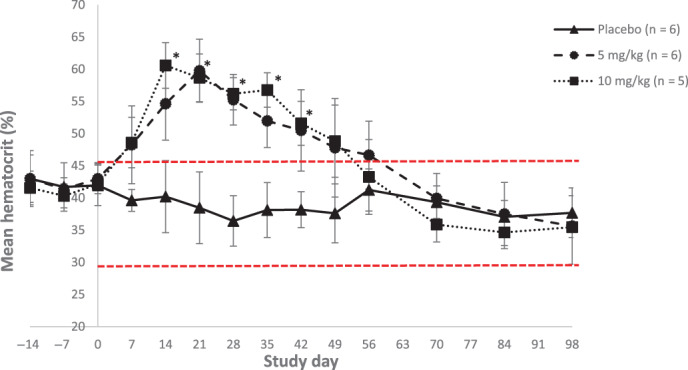
Mean (±SD) hematocrit (%) per group after 0, 5, or 10 mg/kg daily PO administrations of molidustat to healthy adult cats. *Indicates a significant difference between the molidustat‐treated groups compared to the placebo group (*P* < .0002). Red lines represent the lower and upper hematocrits of the normal physiological range for cats (29%‐45%). In molidustat‐treated groups, HCT exceeded 60% on Day 21 (5 mg/kg; Group 2) and Day 14 (10 mg/kg; Group 3) when treatment was stopped.

Compared to placebo (mean HCT range, 39‐42%), a significant increase in mean HCT was evident starting on Day 14 (Group 2:54.4% vs 40.3%, *P* < .001, 95% confidence interval [CI] for the difference [8.95‐19.28]; Group 3:61.2% vs 40.3%, *P* < .001, 95% CI [15.48‐26.43]) and remained significantly higher for the entire treatment period (Figure 1).

**FIGURE 2:**
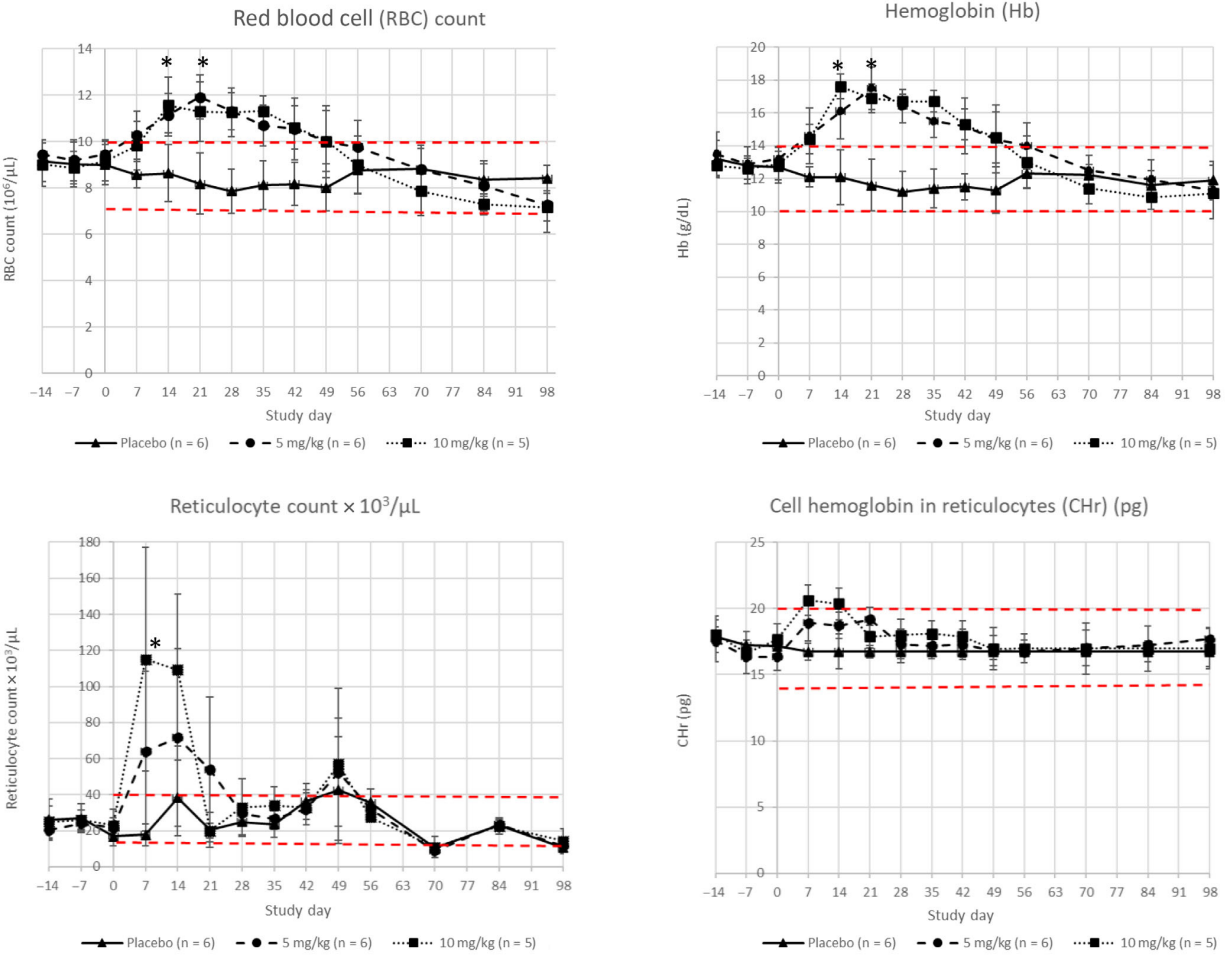
Mean (±SD) of hematological parameters (RBC count, hemoglobin [Hb], total reticulocyte count and Hb in reticulocytes [CHr]) in healthy cats treated PO with 0 (triangles), 5 (circles) or 10 (squares) mg/kg of molidustat daily for 23, 23 and 15 days, respectively. Physiological reference ranges in cats are 7‐10 × 10^6^/μL, 10‐14 g/dL, 15‐40 × 10^3^/μL, and 14.0‐19.9 pg for RBC count, Hb, total reticulocyte count, and CHr, respectively. *Indicates a significant difference between molidustat‐treated groups compared to placebo (*P* < .05) assessed on Days 7, 14, and 21 for RBC count, Hb, and total reticulocyte count. Red lines represent lower and upper physiological ranges.

Mean HCT in molidustat‐treated cats returned to within the reference range (29%‐45%) after Day 56 and results were numerically comparable to those of the placebo group from Day 70 onward. Similar results were recorded for mean RBC counts, Hb concentrations and reticulocyte counts, whereas CHr remained within the physiological range of 14.0 to 19.9 pg^10^ throughout the study (Figure 2).

**FIGURE 3:**
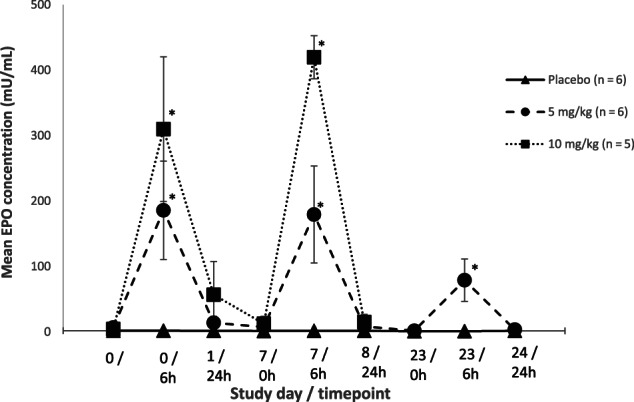
Mean (±SD) erythropoietin (EPO) concentrations (mU/mL) per group after 0, 5, or 10 mg/kg daily PO administrations of molidustat to healthy adult cats. *Indicates a significant difference between the molidustat‐treated groups compared to the placebo group (*P* < .05).

Mean EPO concentrations significantly differed in both molidustat‐treated groups 6 hours after treatment compared to the placebo group on all assessment days (*P* < .05; Figure 3). Mean EPO concentrations ranged from 79 to 186 (on Days 0, 7 and 23) and 310 to 420 (on Days 0 and 7) mU/mL after 5 and 10 mg/kg administrations, respectively, compared to a mean range of 1 to 2 (Days 0, 7 and 23) mU/mL for placebo.

**FIGURE 4:**
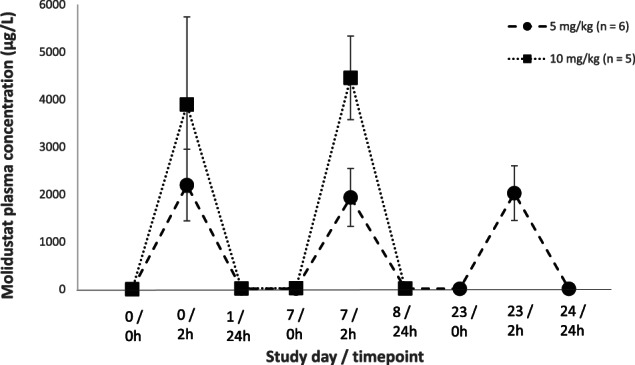
Mean (±SD) plasma molidustat concentrations (μg/L) per group after 5 or 10 mg/kg daily PO administrations of molidustat to healthy adult cats.

Mean plasma molidustat concentrations in Group 2 (5 mg/kg) ranged from 1936 to 2196 μg/L at 2 hours after treatment and 10 to 17 μg/L at 24 hours after treatment (Figure 4). In Group 3 cats, plasma molidustat concentrations ranged from 3894 to 4456 μg/L 2 hours after administration.

Abnormal clinical findings observed in individual cats during physical examinations were mild and considered unlikely to be treatment related (Supplemental Table A). Mean BW did not change with molidustat treatments (Supplemental Table B), nor did food consumption. Fecal consistency did not change throughout the study except for 1 cat in Group 3 over 3 consecutive days (Supplemental Table A). A decrease in mean white blood cell (WBC) count and mean absolute neutrophil number slightly below the reference interval were observed in Group 3 cats on Day 14 (Supplemental Table C). Because of early cessation of the treatment phase, blood for serum biochemistry was collected on Day 12 (Group 3) and Day 23 (Group 1 and Group 2) rather than Day 56. No abnormal findings were detected in biochemical profiles in any cats (Supplemental Table D).

Treatment‐related vomiting occurred in cats of all groups throughout the treatment period. Because cats were group‐housed, it was not always possible to assign the event to an individual cat. The total number of vomiting events was 1, 5 and 7 for Groups 1, 2 and 3, respectively. When compared to the total number of treatments administered per group, these results correspond to a vomiting rate of <10% for all groups (0.7% for placebo group, 3.5% for 5 mg/kg group and 8.8% for 10 mg/kg group). The number of cats with at least 1 vomiting event was higher in the molidustat‐treated groups (4 of 5/6) compared to placebo (1 of 6).

## 4 DISCUSSION

This randomized prospective study conducted in healthy adult cats confirmed the anticipated pharmacodynamic effects of molidustat, a novel HIF‐PH inhibitor, in stimulating production of EPO and consequently increasing hematologic parameters such as HCT, RBC counts, Hb concentrations and reticulocyte counts in a dose‐dependent manner after once daily dosing. These effects were already observed at the first measured timepoint (Day 7) with significance starting on Day 14 when compared to placebo. Results also showed that rapid and marked increases (≥60%) above normal physiological reference ranges of HCT occurred in a dose‐dependent manner after 2 and 3 weeks of daily PO administrations of a suspension of molidustat at 10 mg/kg BW and 5 mg/kg BW, respectively, in healthy cats. Residual effects on HCT in healthy cats after the last molidustat administration were noted up to 42 days for the 10 mg/kg group and 35 days for the 5 mg/kg group.

Hypoxia‐inducible factors (HIFs) are key regulators of proteins involved in erythropoiesis, angiogenesis and glycolysis implicated in the homeostatic adaptation to changes in cellular oxygen content and controlled by HIF‐PH enzymes.^1^ Because HIF‐PH enzymes use oxygen and 2‐oxoglutarate as substrates, their activity is controlled by changes in oxygen concentrations.^1^, ^3^, ^4^, ^5^, ^11^ By reversibly inhibiting HIF‐PH, molidustat emulates hypoxic conditions in the kidney thereby stabilizing the HIF complex and stimulating endogenous EPO production.^1^, ^2^, ^3^ Subsequent erythropoietic responses result in increases in RBC counts, HCT and Hb concentrations as observed in our study.

The influence of molidustat on iron storage within reticulocytes was evaluated by measuring CHr, a novel marker reflecting iron‐limited erythropoiesis when values are low.^10^, ^12^ Reticulocyte hemoglobin concentration results from our study showed that daily administration of molidustat did not cause iron deficiency in treated cats, despite increased RBC counts, Hb concentrations and reticulocyte counts. However, treatment was administered to healthy young cats for a short period of time and discontinued as soon as a substantial increase in HCT was observed. A potential reason for the mild decrease in WBC count and neutrophils observed in Group 3 cats could be a physiologic change in the bone marrow microenvironment because of iatrogenic stimulation of RBC production, resulting in a lineage commitment more toward erythropoiesis rather than granulopoiesis in healthy cats. Another possibility would be a hemodynamic shift of neutrophils to the marginal intravascular pool because of the erythrocytosis.

Based on previous exploratory pharmacokinetic (PK) studies, blood samples for analysis of anticipated peak plasma molidustat and EPO concentrations were collected 2 and 6 hours after administration, respectively. Results indicated little to no accumulation of molidustat or EPO plasma concentrations with daily administration of 5 to 10 mg/kg in healthy cats. A lower mean EPO concentration was observed for the 5 mg/kg group on Day 23. Based on the time frame of our study, it could not be concluded if this change was a true decrease or within physiological variation. In previously conducted exploratory PK studies, molidustat was excreted from feline plasma with an elimination half‐life (t_1/2_) of 4 to 6 hours after PO administration, supporting once daily administration of this HIF‐PH inhibitor stimulating a pulsatile effect on EPO rather than a constant maintenance of high systemic concentrations of EPO (internal communication). This observation further validates the safety profile of molidustat compared to currently available erythropoietin stimulating agents (ESAs). Because renal excretion is negligible, molidustat is also favorable for patients with impaired renal function and thus adjustments in the dosage regimen are not expected for CKD cats treated with molidustat.

Limitations of our study include a limited number of data points for PK and EPO plasma concentrations which would have allowed for pharmacokinetic and pharmacodynamic modeling, a low number of cats, the innate bias of administering molidustat to cats with healthy kidneys, and non‐validated use of EPO test kits designed for humans which may not adequately measure EPO in cats (despite 85% homology). Additionally, vomiting could not be assigned to individual cats and therefore complete analysis of vomiting events was not possible.

To date, ESAs, such as recombinant human EPO (epoetin and darbepoetin), currently are used as off‐label EPO replacements for anemic cats.^7^, ^8^, ^11^ This parenteral treatment has been an important tool in the management of anemia in cats, substantially improving their quality of life. However, numerous complications such as iron deficiency, hypertension, arthralgia, fever, seizures, polycythemia, and life‐threatening pure red cell aplasia are reported in treated cats.^8^, ^11^ A novel and safe approach to manage anemia in cats therefore is needed.

Our objectives of investigating the ability of molidustat to stimulate erythropoiesis in the healthy feline kidney were fulfilled. Once daily administrations resulted in marked pulsatile increases in EPO concentration compared to endogenous concentrations (100‐250×) 6 hours after administration of molidustat. This increase in EPO precipitated a significant increase in HCT above the physiological reference range in healthy cats, confirming its pharmacodynamic efficacy despite normoxic conditions. In our study, HCT was confirmed as an appropriate surrogate for monitoring EPO activity. Hemotocrit is easy to assess and monitor in a clinical setting as part of hematological testing already conducted by veterinarians. Therefore, the postulated concept that a HIF‐PH inhibitor such as molidustat might be well suited for the induction of endogenous EPO production by mimicking hypoxia in the feline kidney was proven. Additional studies to confirm its effects in anemic cats with CKD are warranted.

## CONFLICT OF INTEREST DECLARATION

Drs A. Boegel, I. Flamme, R. Krebber, F. Schmidt, E. Kruedewagen, S. Manfold‐Gehring and G. Beddies, and T. Settje are or were employed by Elanco. Dr C. Lainesse received payments as an independent consultant.

## OFF‐LABEL ANTIMICROBIAL DECLARATION

Authors declare no off‐label use of antimicrobials.

## INSTITUTIONAL ANIMAL CARE AND USE COMMITTEE (IACUC) OR OTHER APPROVAL DECLARATION

Conducted in accordance with local animal welfare laws and regulations (Directive 2010/63/EU, German animal protection act and German animal welfare regulation) as well as company guidelines and procedures. The protocol was approved by responsible authorities (LANUV‐Landesamt fuer Natur, Umwelt und Verbraucherschutz).

## HUMAN ETHICS APPROVAL DECLARATION

Authors declare human ethics approval was not needed for this study.

## Supporting information


**Supplemental Table A:** Abnormal clinical findings during physical examinations.

**Supplemental Table B:** Summary statistics for body weights (kg).

**Supplemental Table C:** Summary statistics for white blood cell parameters.

**Supplemental Table D:** Summary statistics for clinical chemistry.
