# Supplementary material for: Pharmacodynamic effects of molidustat on erythropoiesis in healthy cats
Source: J Vet Intern Med. 2023 Nov 23;38(1):381–7. doi: 10.1111/jvim.16827 (PMC10800175; doi:10.1111/jvim.16827)
Supplement: Supplementary file 1 — Supplemental Table A: Abnormal clinical findings during physical examinations. [file JVIM-38-381-s002.pdf]

Supplemental Table A: Abnormal Clinical Findings during Physical Examinations

Group	Animal ID	Study Day	Abnormality
1	OQO3	-1	Right corner of the mouth slightly swollen and reddened. Suspicion of eosinophilic granuloma. Repeated sneezing when thorax is touched
	HQF1	9	Mild lameness in right forelimb
2	OQB1	13	Fractious
		27	Fractious
3	HMJ1	-1	Fractious, very slim
		9-11	Soft, smelly feces
	HPN1	13	Ulceration around the anus
	ONS4	-1	Anxious
		69	Alopecia left lumbosacral area due to licking
		83	Still alopecia (see SD 69)
		97	Alopecia smaller than last week, hair is growing
	OPW2	-1	Slim
