# Supplementary material for: Pharmacodynamic effects of molidustat on erythropoiesis in healthy cats
Source: J Vet Intern Med. 2023 Nov 23;38(1):381–7. doi: 10.1111/jvim.16827 (PMC10800175; doi:10.1111/jvim.16827)
Supplement: Supplementary file 2 — Supplemental Table B: Summary statistics for body weights (kg). [file JVIM-38-381-s004.pdf]

Supplemental Table B: Summary Statistics for Body Weights (kg)

Group	Statistic	Day -14	Day -7	Day -1	Day 6	Day 13	Day 20	Day 27	Day 34	Day 41	Day 48	Day 55	Day 69	Day 83	Day 97
1	Mean	4.40	4.52	4.45	4.53	4.55	4.52	4.55	4.60	4.55	4.48	4.55	4.42	4.42	4.48
	SD	0.815	0.808	0.817	0.826	0.817	0.794	0.804	0.754	0.787	0.778	0.784	0.880	0.857	0.952
	N	6	6	6	6	6	6	6	6	6	6	6	6	6	6
2	Mean	4.30	4.38	4.30	4.37	4.33	4.30	4.32	4.38	4.33	4.28	4.37	4.23	4.33	4.37
	SD	1.156	1.172	1.114	1.150	1.150	1.182	1.123	1.065	1.127	1.105	1.044	1.129	1.102	1.157
	N	6	6	6	6	6	6	6	6	6	6	6	6	6	6
3	Mean	3.90	3.92	3.90	4.02	3.98	3.98	4.02	4.04	3.98	3.90	3.96	3.94	3.92	4.00
	SD	0.561	0.581	0.570	0.531	0.526	0.526	0.540	0.451	0.502	0.453	0.445	0.434	0.507	0.480
	N	5	5	5	5	5	5	5	5	5	5	5	5	5	5
