# Supplementary material for: Pharmacodynamic effects of molidustat on erythropoiesis in healthy cats
Source: J Vet Intern Med. 2023 Nov 23;38(1):381–7. doi: 10.1111/jvim.16827 (PMC10800175; doi:10.1111/jvim.16827)
Supplement: Supplementary file 3 — Supplemental Table C: Summary statistics for white blood cell parameters. [file JVIM-38-381-s001.pdf]

Supplemental Table C: Summary Statistics for White Blood Cell Parameters

Parameter	Group	Statistic	Day -14	Day -7	Day 0	Day 7	Day 14	Day 21	Day 28	Day 35	Day 42	Day 49	Day 56	Day 70	Day 84	Day 98
WBC [10^3/µL] L=6.3, H=19.6	1	Mean	10.61	11.03	9.63	9.68	9.19	10.87	10.98	12.83	11.69	12.16	12.02	12.77	12.62	14.54
		SD	2.721	3.027	2.593	2.984	2.254	3.773	3.050	3.597	2.402	3.302	4.449	4.811	3.355	8.289
		N	6	6	6	6	6	6	6	6	6	6	6	6	6	6
	2	Mean	8.48	9.49	8.52	6.83	6.73	7.32	8.23	10.40	11.20	10.50	9.74	9.16	8.39	9.06
		SD	0.860	1.448	1.546	2.003	0.945	1.133	1.466	1.396	0.699	1.442	0.976	1.246	1.552	1.609
		N	6	6	6	6	6	6	6	6	6	6	6	6	6	6
	3	Mean	9.73	10.85	10.20	8.38	6.20	8.52	9.89	13.81	12.35	12.28	12.04	10.72	12.28	10.17
		SD	1.578	1.501	1.097	0.523	0.277	1.456	2.365	2.341	2.266	1.950	2.659	2.132	4.748	1.819
		N	5	5	5	5	5	5	5	5	5	5	5	5	5	5
NEUT [#] L=3.0, H=13.4	1	Mean	4.20	4.89	4.02	4.29	3.68	4.53	5.04	5.64	5.37	5.30	5.40	6.77	6.88	9.04
		SD	1.217	1.826	1.624	2.072	1.099	2.344	1.417	2.087	1.465	1.949	2.924	4.072	2.130	9.055
		N	6	6	6	6	6	6	6	6	6	6	6	6	6	6
	2	Mean	3.74	5.06	4.17	3.22	3.27	3.97	4.29	5.51	6.03	4.80	4.63	4.16	4.05	4.78
		SD	0.570	1.021	1.402	1.580	0.988	0.566	0.543	1.427	1.223	1.385	1.109	1.161	0.877	1.410
		N	6	6	6	6	6	6	6	6	6	6	6	6	6	6
	3	Mean	3.72	4.62	3.89	3.77	2.34	3.53	4.55	5.24	5.40	4.00	4.30	4.73	6.02	3.91
		SD	0.971	1.317	0.880	1.099	0.348	1.326	2.401	2.237	1.678	1.676	1.968	1.864	4.706	1.376
		N	5	5	5	5	5	5	5	5	5	5	5	5	5	5
NEUT [%] L=29.5, H=74.5	1	Mean	40.48	44.00	41.57	43.70	40.43	40.88	46.27	43.87	46.15	43.70	43.32	50.08	54.15	54.03
		SD	10.757	9.316	11.974	10.911	8.669	11.088	5.145	14.599	10.571	11.563	11.723	13.226	6.490	19.582
		N	6	6	6	6	6	6	6	6	6	6	6	6	6	6
	2	Mean	44.42	53.23	48.47	46.07	48.50	54.45	52.67	52.40	53.77	45.78	47.72	45.48	48.02	52.47
		SD	7.547	6.548	10.815	11.379	11.177	5.237	4.019	8.639	10.315	12.185	10.907	10.794	4.019	9.445
		N	6	6	6	6	6	6	6	6	6	6	6	6	6	6
	3	Mean	37.88	42.16	38.00	44.84	37.80	41.04	44.06	37.82	43.56	32.30	34.72	42.72	45.08	38.26
		SD	5.018	8.050	6.431	11.826	6.033	12.730	12.026	12.987	7.999	10.051	7.846	9.912	15.543	9.516
		N	5	5	5	5	5	5	5	5	5	5	5	5	5	5

L=reference range lower limit, H=reference range upper limit, SD=standard deviation, N=sample size

Supplemental Table C: Summary Statistics for White Blood Cell Parameters

Parameter	Group	Statistic	Day -14	Day -7	Day 0	Day 7	Day 14	Day 21	Day 28	Day 35	Day 42	Day 49	Day 56	Day 70	Day 84	Day 98
BASO [#]	1	Mean	0.04	0.03	0.03	0.03	0.03	0.04	0.03	0.04	0.03	0.04	0.04	0.04	0.05	0.07
L=0.0, H=0.1		SD	0.018	0.013	0.014	0.013	0.010	0.010	0.015	0.019	0.008	0.022	0.023	0.026	0.028	0.044
		N	6	6	6	6	6	6	6	6	6	6	6	6	6	6
	2	Mean	0.02	0.03	0.02	0.02	0.02	0.03	0.03	0.03	0.04	0.04	0.02	0.03	0.02	0.03
		SD	0.010	0.005	0.006	0.005	0.010	0.010	0.008	0.009	0.027	0.022	0.015	0.013	0.008	0.013
		N	6	6	6	6	6	6	6	6	6	6	6	6	6	6
	3	Mean	0.03	0.03	0.03	0.03	0.03	0.04	0.04	0.06	0.05	0.05	0.05	0.04	0.04	0.04
		SD	0.013	0.008	0.004	0.009	0.009	0.005	0.011	0.015	0.013	0.005	0.011	0.011	0.012	0.015
		N	5	5	5	5	5	5	5	5	5	5	5	5	5	5
BASO [%]	1	Mean	0.32	0.27	0.23	0.27	0.28	0.33	0.25	0.32	0.25	0.33	0.32	0.28	0.33	0.43
L=0.0, H=1.0		SD	0.041	0.103	0.103	0.103	0.041	0.082	0.084	0.075	0.055	0.121	0.075	0.075	0.151	0.082
		N	6	6	6	6	6	6	6	6	6	6	6	6	6	6
	2	Mean	0.25	0.28	0.25	0.30	0.32	0.35	0.32	0.28	0.32	0.33	0.23	0.35	0.28	0.32
		SD	0.084	0.041	0.105	0.126	0.098	0.084	0.041	0.075	0.240	0.137	0.151	0.122	0.041	0.147
		N	6	6	6	6	6	6	6	6	6	6	6	6	6	6
	3	Mean	0.32	0.28	0.28	0.34	0.50	0.44	0.38	0.40	0.38	0.40	0.42	0.38	0.36	0.40
		SD	0.084	0.084	0.045	0.055	0.122	0.055	0.164	0.071	0.084	0.000	0.084	0.084	0.055	0.122
		N	5	5	5	5	5	5	5	5	5	5	5	5	5	5

L=reference range lower limit, H=reference range upper limit, SD=standard deviation, N=sample size

Supplemental Table C: Summary Statistics for White Blood Cell Parameters

Parameter	Group	Statistic	Day -14	Day -7	Day 0	Day 7	Day 14	Day 21	Day 28	Day 35	Day 42	Day 49	Day 56	Day 70	Day 84	Day 98
EOS [#]	1	Mean	0.91	0.81	0.69	0.71	1.03	1.24	0.99	1.42	1.24	1.42	1.22	0.85	0.71	0.74
L=0.3, H=1.7		SD	0.793	0.270	0.298	0.279	0.386	0.673	0.418	0.903	0.640	0.799	0.577	0.341	0.341	0.529
		N	6	6	6	6	6	6	6	6	6	6	6	6	6	6
	2	Mean	0.54	0.53	0.55	0.43	0.58	0.54	0.66	1.00	0.92	1.15	0.86	0.70	0.60	0.56
		SD	0.267	0.246	0.174	0.233	0.329	0.164	0.235	0.294	0.296	0.253	0.561	0.396	0.295	0.279
		N	6	6	6	6	6	6	6	6	6	6	6	6	6	6
	3	Mean	0.71	0.83	0.74	0.50	0.34	0.58	0.90	1.07	0.91	1.41	1.09	0.54	0.84	0.71
		SD	0.406	0.515	0.334	0.227	0.198	0.152	0.113	0.582	0.437	0.610	0.526	0.305	0.549	0.523
		N	5	5	5	5	5	5	5	5	5	5	5	5	5	5
EOS [%]	1	Mean	8.35	7.85	7.42	7.65	11.00	11.03	8.82	10.37	10.07	10.87	10.07	6.67	5.48	5.93
L=3.4, H=11.4		SD	6.738	3.149	3.156	2.958	2.571	4.666	2.207	4.506	3.796	4.628	2.973	1.334	1.572	4.711
		N	6	6	6	6	6	6	6	6	6	6	6	6	6	6
	2	Mean	6.50	5.42	6.57	6.78	8.73	7.57	8.27	9.80	8.20	11.03	8.65	7.32	6.97	6.25
		SD	3.623	1.905	2.252	4.882	5.272	3.158	3.351	3.668	2.753	2.428	5.241	3.947	3.057	3.361
		N	6	6	6	6	6	6	6	6	6	6	6	6	6	6
	3	Mean	7.24	7.56	7.26	5.96	5.60	7.18	9.38	7.46	7.12	11.24	8.78	5.34	6.40	6.52
		SD	3.902	4.570	3.021	2.598	3.198	2.787	1.638	3.251	2.662	4.328	2.982	3.332	2.750	3.743
		N	5	5	5	5	5	5	5	5	5	5	5	5	5	5

L=reference range lower limit, H=reference range upper limit, SD=standard deviation, N=sample size

Supplemental Table C: Summary Statistics for White Blood Cell Parameters

Parameter	Group	Statistic	Day -14	Day -7	Day 0	Day 7	Day 14	Day 21	Day 28	Day 35	Day 42	Day 49	Day 56	Day 70	Day 84	Day 98
LYMPH [#]	1	Mean	5.25	5.05	4.71	4.41	4.22	4.81	4.66	5.41	4.80	5.16	5.07	4.86	4.65	4.30
L=2.0, H=7.2		SD	1.864	1.662	1.581	1.619	1.391	1.493	1.430	1.749	1.275	1.559	1.460	1.182	1.237	1.815
		N	6	6	6	6	6	6	6	6	6	6	6	6	6	6
	2	Mean	4.02	3.65	3.60	2.95	2.61	2.52	3.00	3.56	4.01	4.32	4.02	4.04	3.47	3.43
		SD	1.053	0.763	0.878	0.984	0.819	0.817	0.944	0.845	1.217	1.671	1.204	1.156	0.683	0.929
		N	6	6	6	6	6	6	6	6	6	6	6	6	6	6
	3	Mean	5.04	5.16	5.34	3.84	3.31	4.13	4.16	7.18	5.73	6.59	6.34	5.15	5.04	5.16
		SD	0.528	0.983	0.897	0.999	0.423	1.235	0.903	2.122	1.127	1.274	0.878	0.446	1.204	1.145
		N	5	5	5	5	5	5	5	5	5	5	5	5	5	5
LYMPH [%]	1	Mean	48.77	45.58	48.75	45.70	45.70	45.33	42.23	42.80	41.40	43.08	43.95	40.88	37.40	36.77
L=20.0, H=61.2		SD	5.810	7.546	10.202	10.049	8.787	9.156	5.323	14.335	10.798	11.101	10.108	12.579	5.401	17.015
		N	6	6	6	6	6	6	6	6	6	6	6	6	6	6
	2	Mean	46.90	38.67	42.55	43.40	38.55	33.82	35.68	34.58	35.78	40.92	41.33	44.38	41.73	38.07
		SD	8.837	7.087	10.309	12.251	10.105	6.379	5.520	8.009	10.345	12.850	11.553	11.354	6.070	9.950
		N	6	6	6	6	6	6	6	6	6	6	6	6	6	6
	3	Mean	52.24	48.08	52.58	45.96	53.22	48.46	43.72	52.48	46.96	54.26	54.04	49.22	45.42	51.64
		SD	3.860	9.287	8.300	12.342	5.089	11.714	11.430	13.677	8.165	9.568	9.292	8.219	15.920	11.648
		N	5	5	5	5	5	5	5	5	5	5	5	5	5	5

L=reference range lower limit, H=reference range upper limit, SD=standard deviation, N=sample size

Supplemental Table C: Summary Statistics for White Blood Cell Parameters

Parameter	Group	Statistic	Day -14	Day -7	Day 0	Day 7	Day 14	Day 21	Day 28	Day 35	Day 42	Day 49	Day 56	Day 70	Day 84	Day 98
MONO [#]	1	Mean	0.21	0.25	0.19	0.24	0.23	0.25	0.25	0.32	0.25	0.23	0.28	0.25	0.32	0.38
L=0.0, H=1.0		SD	0.058	0.100	0.068	0.091	0.092	0.109	0.070	0.114	0.073	0.066	0.141	0.085	0.134	0.222
		N	6	6	6	6	6	6	6	6	6	6	6	6	6	6
	2	Mean	0.16	0.21	0.18	0.22	0.25	0.27	0.24	0.29	0.21	0.20	0.20	0.22	0.24	0.25
		SD	0.031	0.046	0.040	0.056	0.087	0.053	0.037	0.118	0.052	0.078	0.073	0.048	0.053	0.062
		N	6	6	6	6	6	6	6	6	6	6	6	6	6	6
	3	Mean	0.22	0.20	0.19	0.23	0.17	0.23	0.23	0.25	0.26	0.23	0.24	0.25	0.33	0.32
		SD	0.083	0.068	0.090	0.066	0.047	0.046	0.068	0.117	0.135	0.101	0.097	0.099	0.169	0.115
		N	5	5	5	5	5	5	5	5	5	5	5	5	5	5
MONO [%]	1	Mean	1.98	2.25	2.00	2.55	2.52	2.37	2.35	2.57	2.10	1.93	2.28	1.97	2.50	2.73
L=0.2, H=5.0		SD	0.436	0.554	0.502	0.568	0.719	0.709	0.558	1.021	0.379	0.524	0.483	0.450	0.603	0.755
		N	6	6	6	6	6	6	6	6	6	6	6	6	6	6
	2	Mean	1.90	2.30	2.13	3.30	3.78	3.73	2.93	2.80	1.82	1.92	2.02	2.38	2.87	2.75
		SD	0.494	0.704	0.388	1.086	1.504	1.054	0.734	0.921	0.527	0.760	0.637	0.578	0.314	0.663
		N	6	6	6	6	6	6	6	6	6	6	6	6	6	6
	3	Mean	2.22	1.80	1.84	2.74	2.76	2.80	2.36	1.74	1.98	1.78	1.94	2.30	2.66	3.00
		SD	0.507	0.458	0.754	0.688	0.673	0.524	0.594	0.594	0.733	0.581	0.541	0.570	0.767	0.604
		N	5	5	5	5	5	5	5	5	5	5	5	5	5	5

L=reference range lower limit, H=reference range upper limit, SD=standard deviation, N=sample size

ABBREVIATIONS
WBC: white blood cells
NEUT: neutrophils
BASO: basophils
EOS: eosinophils
LYMPH: lymphocytes
MONO: monocytes
