# Supplementary material for: Pharmacodynamic effects of molidustat on erythropoiesis in healthy cats
Source: J Vet Intern Med. 2023 Nov 23;38(1):381–7. doi: 10.1111/jvim.16827 (PMC10800175; doi:10.1111/jvim.16827)
Supplement: Supplementary file 4 — Supplemental Table D: Summary statistics for clinical chemistry. [file JVIM-38-381-s003.pdf]

Supplemental Table D: Summary Statistics for Clinical Chemistry

Parameter	Group	Statistic	Day -14	Day 12	Day 23	Day 97
ALB [g/L]	1	Mean	31.83	--	29.50	33.67
L=22, H=40		SD	3.920	--	2.168	1.966
		N	6	--	6	6
	2	Mean	29.83	--	29.83	32.33
		SD	2.639	--	3.488	1.862
		N	6	--	6	6
	3	Mean	30.00	30.60	--	32.40
		SD	2.236	0.894	--	0.548
		N	5	5	--	5
ALB/GLOB	1	Mean	0.80	--	0.80	1.00
		SD	0.089	--	0.089	0.155
		N	6	--	6	6
	2	Mean	0.73	--	0.72	0.95
		SD	0.052	--	0.098	0.055
		N	6	--	6	6
	3	Mean	0.74	0.70	--	0.88
		SD	0.055	0.071	--	0.045
		N	5	5	--	5
ALP [U/L]	1	Mean	40.67	--	36.83	35.17
L=14, H=111		SD	16.452	--	14.511	16.845
		N	6	--	6	6
	2	Mean	37.50	--	33.17	30.17
		SD	9.894	--	7.960	9.475
		N	6	--	6	6
	3	Mean	36.80	37.20	--	37.00
		SD	10.710	13.424	--	13.856
		N	5	5	--	5

L=reference range lower limit, H=reference range upper limit, SD=standard deviation, N=sample size

Supplemental Table D: Summary Statistics for Clinical Chemistry

Parameter	Group	Statistic	Day -14	Day 12	Day 23	Day 97
ALT [U/L]	1	Mean	89.33	--	76.83	64.67
L=12, H=130		SD	22.563	--	19.323	18.811
		N	6	--	6	6
	2	Mean	81.33	--	84.33	81.00
		SD	18.457	--	23.729	32.551
		N	6	--	6	6
	3	Mean	77.00	78.00	--	78.20
		SD	19.634	25.010	--	35.815
		N	5	5	--	5
BUN/CREA	1	Mean	11.33	--	13.83	13.67
		SD	1.366	--	2.137	2.160
		N	6	--	6	6
	2	Mean	11.33	--	13.00	14.00
		SD	1.633	--	1.897	2.683
		N	6	--	6	6
	3	Mean	12.00	10.20	--	11.80
		SD	1.414	2.168	--	1.643
		N	5	5	--	5
Bile acids	1	Mean	0.00	--	0.00	0.00
		SD	0.000	--	0.000	0.000
		N	6	--	6	6
	2	Mean	0.00	--	0.00	0.00
		SD	0.000	--	0.000	0.000
		N	6	--	6	6
	3	Mean	0.00	0.00	--	0.00
		SD	0.000	0.000	--	0.000
		N	5	5	--	5

L=reference range lower limit, H=reference range upper limit, SD=standard deviation, N=sample size

Supplemental Table D: Summary Statistics for Clinical Chemistry

Parameter	Group	Statistic	Day -14	Day 12	Day 23	Day 97
CA [mmol/L]	1	Mean	2.32	--	2.20	2.32
L=1.95, H=2.83		SD	0.139	--	0.103	0.139
		N	6	--	6	6
	2	Mean	2.30	--	2.22	2.30
		SD	0.099	--	0.140	0.094
		N	6	--	5	6
	3	Mean	2.28	2.38	--	2.27
		SD	0.059	0.058	--	0.048
		N	5	5	--	5
CHOL [mmol/L]	1	Mean	4.08	--	3.99	4.74
L=1.68, H=5.81		SD	0.953	--	0.833	1.200
		N	6	--	6	6
	2	Mean	3.82	--	3.73	4.28
		SD	0.773	--	0.673	0.454
		N	6	--	6	6
	3	Mean	3.61	3.76	--	4.74
		SD	0.948	0.736	--	1.135
		N	5	5	--	5
CREA [µmol/L]	1	Mean	144.17	--	126.17	132.33
L=71, H=212		SD	12.734	--	20.999	15.958
		N	6	--	6	6
	2	Mean	148.67	--	154.50	138.67
		SD	15.319	--	23.864	14.431
		N	6	--	6	6
	3	Mean	144.60	199.40	--	147.00
		SD	14.467	25.304	--	7.071
		N	5	5	--	5

L=reference range lower limit, H=reference range upper limit, SD=standard deviation, N=sample size

Supplemental Table D: Summary Statistics for Clinical Chemistry

Parameter	Group	Statistic	Day -14	Day 12	Day 23	Day 97
GLOB [g/L]	1	Mean	39.67	--	37.00	34.00
L=28, H=51		SD	2.251	--	1.265	4.050
		N	6	--	6	6
	2	Mean	40.50	--	41.83	34.00
		SD	4.135	--	3.869	2.966
		N	6	--	6	6
	3	Mean	40.60	43.60	--	36.00
		SD	3.435	2.302	--	2.828
		N	5	5	--	5
GLU [mmol/L]	1	Mean	4.01	--	4.75	4.36
L=4.11, H=8.84		SD	0.532	--	0.477	0.627
		N	6	--	6	6
	2	Mean	3.89	--	3.66	4.10
		SD	0.467	--	0.850	0.419
		N	6	--	6	6
	3	Mean	4.09	3.98	--	4.22
		SD	0.397	0.354	--	0.412
		N	5	5	--	5
NH3 [µmol/L]	1	Mean	0.00	--	0.00	0.00
L=0, H=95		SD	0.000	--	0.000	0.000
		N	6	--	6	6
	2	Mean	0.00	--	0.00	0.00
		SD	0.000	--	0.000	0.000
		N	6	--	6	6
	3	Mean	0.00	0.00	--	0.00
		SD	0.000	0.000	--	0.000
		N	5	5	--	5

L=reference range lower limit, H=reference range upper limit, SD=standard deviation, N=sample size

Supplemental Table D: Summary Statistics for Clinical Chemistry

Parameter	Group	Statistic	Day -14	Day 12	Day 23	Day 97
PHOS [mmol/L]	1	Mean	1.74	--	1.79	1.55
L=1.0, H=2.42		SD	0.112	--	0.173	0.082
		N	6	--	6	6
	2	Mean	1.54	--	1.79	1.46
		SD	0.216	--	0.345	0.256
		N	6	--	5	6
	3	Mean	1.44	1.85	--	1.35
		SD	0.100	0.168	--	0.155
		N	5	5	--	5
TBIL [µmol/L]	1	Mean	5.00	--	3.00	2.75
L=0, H=15		SD	2.449	--	1.414	0.957
		N	5	--	4	4
	2	Mean	4.20	--	4.67	2.75
		SD	1.789	--	2.160	0.500
		N	5	--	6	4
	3	Mean	4.40	6.20	--	2.25
		SD	2.510	0.837	--	0.500
		N	5	5	--	4
TP [g/L]	1	Mean	71.50	--	66.50	67.67
L=57, H=89		SD	5.822	--	1.871	3.386
		N	6	--	6	6
	2	Mean	70.33	--	71.67	66.33
		SD	6.470	--	6.186	4.676
		N	6	--	6	6
	3	Mean	70.60	74.20	--	68.40
		SD	5.413	1.924	--	2.702
		N	5	5	--	5

L=reference range lower limit, H=reference range upper limit, SD=standard deviation, N=sample size

Supplemental Table D: Summary Statistics for Clinical Chemistry

Parameter	Group	Statistic	Day -14	Day 12	Day 23	Day 97
UREA [mmol/L]	1	Mean	6.58	--	7.02	7.10
L=5.7, H=12.9		SD	1.342	--	1.070	0.764
		N	6	--	6	6
	2	Mean	6.82	--	8.05	7.80
		SD	1.009	--	0.579	1.086
		N	6	--	6	6
	3	Mean	6.94	8.26	--	6.94
		SD	0.422	2.707	--	0.873
		N	5	5	--	5

L=reference range lower limit, H=reference range upper limit, SD=standard deviation, N=sample size

ABBREVIATIONS
ALB : albumin
GLOB : globulin
ALP : alkaline phosphatase
ALT : alanine transferase
BUN : blood urea nitrogen
CREA : creatinine
CA: calcium
CHOL: cholesterol
GLU : glucose
NH3 : ammonia
PHOS : phosphorous
TBil: total bilirubin
TP : total protein

Clinical parameters were analyzed in house with a reflectance photometer (Catalyst DX®, IDEXX GmbH, 55286, Wörrstadt, Germany).
